# Effect of α-Amylase Degradation on Physicochemical Properties of Pre-High Hydrostatic Pressure-Treated Potato Starch

**DOI:** 10.1371/journal.pone.0143620

**Published:** 2015-12-07

**Authors:** Tai-Hua Mu, Miao Zhang, Leyla Raad, Hong-Nan Sun, Cheng Wang

**Affiliations:** 1 Institute of Agro-Products Processing Science and Technology, Chinese Academy of Agricultural Sciences; Key Laboratory of Agro-Products Processing, Ministry of Agriculture, Haidian District, Beijing, P. R. China; 2 Institute of Quality Standard and Testing Technology for Agro-Products, Xinjiang Academy of Agricultural sciences, Xinjiang Uygur Autonomous Region, China; Agriculture and Agri-Food Canada, CANADA

## Abstract

The effect of high hydrostatic pressure (HHP) on the susceptibility of potato starch (25%, w/v) suspended in water to degradation by exposure to bacterial α-amylase (0.02%, 0.04% and 0.06%, w/v) for 40 min at 25°C was investigated. Significant differences (*p* < 0.05) in the structure, morphology and physicochemical properties were observed. HHP-treated potato starch (PS) exposed to α-amylase (0.06%, w/v) showed a significantly greater degree of hydrolysis and amount of reducing sugar released compared to α-amylase at a concentration of 0.04% (w/v) or 0.02% (w/v). Native PS (NPS) granules have a spherical and elliptical form with a smooth surface, whereas the hydrolyzed NPS (hNPS) and hydrolyzed HHP-treated PS granules showed irregular and ruptured forms with several cracks and holes on the surface. Hydrolysis of HHP-treated PS by α-amylase could decrease the average granule size significantly (*p* <0.05) from 29.43 to 20.03 μm. Swelling power decreased and solubility increased with increasing enzyme concentration and increasing pressure from 200–600 MPa, with the exception of the solubility of HHP-treated PS at 600 MPa (HHP_600_ PS). Fourier transform infrared spectroscopy (FTIR) showed extensive degradation of the starch in both the ordered and the amorphous structure, especially in hydrolyzed HHP_600_ PS. The B-type of hydrolyzed HHP_600_ PS with α-amylase at a concentration 0.06% (w/v) changed to a B+V type with an additional peak at 2θ = 19.36°. The HHP_600_ starch with 0.06% (w/v) α-amylase displayed the lowest value of *T*_o_ (onset temperature), *T*_c_ (conclusion temperature) and Δ*H*_gel_ (enthalpies of gelatinization). These results indicate the pre-HHP treatment of NPS leads to increased susceptibility of the granules to enzymatic degradation and eventually changes of both the amorphous and the crystalline structures.

## Introduction

Potato (*Solanum tuberosum*) is the third most important food crop in the world after rice (*Oryza sativa*) and wheat (*Triticum aestivum*) in terms of human consumption [1]. China leads the world in potato production, and nearly a third of the world's potatoes are harvested in China and India [2]. Potato starch (PS) is used extensively in the food industry as a coating, blending, bulking and thickening agent. This wide application of starch takes advantage of its desirable flavor and colorless appearance of SP [3]. In terms of physical properties, PS is unique among commercially available starches (e.g. cereal type) and is used widely in a variety of food systems [4]. This uniqueness is due to the large granule size, purity, relatively long amylose and amylopectin chains, the existence of phosphate ester groups on amylopectin and the ability to form thick visco-elastic gels upon heating and subsequent cooling [5].

In general, native starches have an intrinsic imperfect nature, are insoluble in water and tend to retrograde and undergo syneresis, producing weak, cohesive, rubbery pastes when heated and undesirable gels when the pastes are cooled [6], which restricts their use in the food industry. Various modification processes, including physical, chemical, enzymatic and biotechnological, are used to improve the properties and functionality of starch, paying attention to imperfect intrinsic characteristics, water insolubility, tendency to retrograde and undergo syneresis to make it suitable for many food and industrial applications. Physical modification methods are, in general, safe, inexpensive and do not involve any chemical treatment potentially harmful to human health. HHP treatment provides the possibility of making some products with higher nutritional value, more desirable sensory properties and novel texture [7]. There is vast interest in the use of HHP for physical modification of starches, since HHP-gelatinized starch has different properties compared to heat-gelatinized starch [8]. The structure of starch can be altered by treatment with HHP [9]. Reversible hydration of the amorphous phase is followed by irreversible distortion of the crystalline region; the secondary and tertiary structures are broken but the covalent bonds are unaffected [10]. The extent of gelatinization achieved by treatment with HHP is highly dependent on the type of starch, water content, pressure level, temperature range and length of treatment time [11]. Homogenously partially gelatinized starch with a branched star-like structure is one of the characteristics of HHP-treated starch [12], which could be used as transport and delivery vehicles for substances of interest in the cosmetic, food and pharmaceutical industries. In addition, the use of enzymatic modification of starch gives high yields of more specific hydrolysis products and allows better control of the process and the nature of the end products [13]. This process achieves desired functional properties, which extends their range of applications in food and non-food industries, including the manufacture of pharmaceutical, paper and textile products [14] However, no study of the effect of α-amylase treatment on the structure and physicochemical properties of HHP-treated PS has been reported.

The objectives of this study were to evaluate the effect of treatment with HHP (200, 400 and 600 MPa) on the susceptibility of PS to α-amylase hydrolysis and to determine the morphology, thermal properties and crystal properties of native, HHP-treated and hydrolyzed HHP-treated PS. This knowledge will provide a better understanding of modified PS as a functional agent in industry.

## Materials and Methods

### Materials

A Chinese potato cultivar (Qingshu no. 9) frequently used for industrial starch production was selected for this study. Qingshu no. 9 was grown in North Eastern China. Bacterial α-amylase (A4551; activity of 570 units mg^–1^) was purchased from Sigma Chemical Co., Ltd. (Saint Louis, MO, USA). All other chemicals used in this study were of analytical grade.

### Starch isolation

Fresh potatoes were washed, cut into small pieces, immersed in 0.1% (w/v) sodium bisulfite (1 kg of tuber in 1 L of solution) for 10 min and then blended in a domestic juice extractor for 3–4 min. The resulting slurry was passed through a fine muslin cloth to separate the cell debris as described by Peshin [15]. The suspension was filtered three times using a 150 mm mesh size sieve and left to settle overnight at room temperature. The precipitated starch was spread onto oven trays lined with aluminum foil and dried in a hot air dryer (DGG-9240B, Senxin Instruments, Shanghai, China) at 45°C for 24 h. The dried starch was finely ground for 30 s to get powder (FW100 high-speed universal hand mill; TaiSiTe Instrument, Tianjin, China), packaged in tightly covered polypropylene containers and stored at 4°C. The native PS (NPS) was composed of 93.00% (w/w) starch [16] method 996.11, consisting of 19.40% (w/w) amylose and 81.60% (w/w) amylopectin [17], 0.10% (w/w) protein [16] method 955.04, 0.25% (w/w) total lipids [16] method 960.39, 0.03% (w/w) phosphorus [18], 0.33% (w/w) ash[16] method 923.03 and 3.30% (w/w) moisture.

### Treatment with HHP

Treatment with HHP was done in a discontinuous HHP machine (HPP.L3-600/0.6, HuaTai SenMiao Biotechnology Co., Ltd., Tianjin, China) at a pressure of 600 MPa in a steel vessel of 0.6 L capacity (60 mm × 210 mm). A mixture of starch (25%, w/w) and water in a polymer pouch was placed into the vessel containing water as the pressure medium. The temperature inside the vessel was controlled at 25°C by a circulating thermostatically controlled water bath. In each experiment, pressure was increased at a rate of ~100 MPa min^–1^ and was kept at 200, 400 or 600 MPa for 40 min, which were optimized by single-factor experiment (data not shown). After each treatment, the pressure was decreased to atmospheric pressure at ~100 MPa min^–1^, the starch was decanted, freeze-dried and stored in a desiccator. Control starch (NPS) was treated at atmospheric pressure (0.1 MPa) at room temperature (~ 25°C).

### Starch hydrolysis

The starch samples were hydrolyzed with bacterial α-amylase, in duplicate, essentially as described by Franco and Ciacco [19] but with some modification. The starches (25%, w/w) were dispersed in sodium phosphate buffer (0.2 M pH 6.0) with 3 mL of different concentrations (0.02%, 0.04% and 0.06%, w/v) of bacterial α-amylase. Sodium azide (1 mL of 10%, w/v) was added to prevent microbial growth. The starch dispersions were incubated at 20°C for 48 h in an orbital shaker, of which the hydrolysis time were optimized by single-factor experiment (data not shown). Samples of the supernatant were removed at the end of each hydrolysis period and the amount of reducing sugars was measured using 3,5-dinitrosalicylic acid [20]. The hydrolyzed starch slurry were inactivated by adding 0.1 M HCl to achieve pH 3, stirred for 15 min, neutralized by the addition of 0.1 M NaOH and centrifuged at 2000 *g* for 15 min. The hydrolysis residue was washed with water and ethanol. The hydrolyzed starch obtained was air-dried in a forced-air oven at 50°C for 24 h. The percentage (dry weight basis) of hydrolysis was calculated as follows [21]:

$$Hydrolysis\;=\;100\times \left[(W_{0}-W_{1})\;/\;W_{0}\right]\%$$

where *W*_0_ is the weight (dry basis) of untreated starch and *W*_1_ is the weight (dry basis) of hydrolyzed starch.

### Scanning electron microscopy (SEM)

The starch granules were observed using a scanning electron microscope (Hitachi S-3400N, Tokyo, Japan). The starch samples were sprinkled onto double-sided tape mounted on an SEM stub, coated with gold and placed into the SEM chamber. Photomicrographs were taken using an SEM apparatus at an accelerating voltage of 15 kV.

### Particle size

Particle size distribution of the starch granules was measured with a laser particle size analyzer (BT-9300H, Dandong Bettersize™ Instruments Ltd., Dandong, China). The mean granule size of each starch sample was calculated as the mean volume diameter (*D*_50_).

### Differential scanning calorimetry (DSC)

Thermal properties of the starches were analyzed using a differential scanning calorimeter (Q200 TA Instruments, New Castle, DE) essentially as described by Kaur et al. [22] but with slight modification. A sample of starch (2–5 mg) was weighed into an aluminum pan and the appropriate volume of water was added (final mix 1:3, w/w). The pan was sealed hermetically and equilibrated at room temperature for 2 h before the analysis. The DSC instrument was calibrated using indium and an empty pan as the reference. Samples were heated at 10°C min^–1^ over a temperature range of 30°C–120°C. Peak temperature (*T*_p_); onset gelatinization temperature (*T*_o_); conclusion temperature (*T*_c_) and enthalpy of gelatinization (Δ*H*_gel_) were recorded. The gelatinization temperature range (*R*) was calculated as 2(*T*_p_−*T*_o_) [23]

### Fourier transform infrared spectroscopy (FTIR)

The FTIR spectra of the native and hydrolyzed HHP-treated starches were obtained with an IR spectrophotometer (Tensor 37, Bruker Opticals Company, Germany). Starch samples were blended with KBr powder and pressed into thin films before the measurement.

### X-ray diffraction (XRD)

The XRD patterns of native and hydrolyzed HHP-treated starches were collected with X’pert PRO (PANalytical B.V., Holland) according to the modified method described by Chang et al. [24]. The diffractometer was operated at 40 mA and 40 kV. Signals of the reflection angle 2θ from 5°–40° at a scanning rate of 0.21° s^–1^ were recorded.

### Swelling power and solubility

Swelling power and solubility of the starches were determined essentially as described Crosbie [25] but with slight modification. A sample (1.0 ± 0·001 g, dry basis) was weighed into a 50 mL centrifuge tube and distilled water (30 mL) was added from a rapid dispensing pipette. The screw cap was fastened onto the tube immediately and the contents mixed in a vortex mixer; any delay at this stage caused the starch to form lumps. The tube was placed into a constant temperature water bath at 92.5°C ± 0.5°C for 30 min with mixing. The resulting slurry was cooled to room temperature and centrifuged at 2000 *g* for 15 min. The supernatant was decanted into an evaporating dish and dried at 105°C for 5 h. The dried supernatant and the residue were weighed. The solubility (*S*, %) and swelling power (*SP*, g g^–1^, dry basis) were calculated as follows:

$$S\;=\;\begin{array}{cccc} Weight & of & dried & \text{sup}\;erna\;\text{tan}\;t/\begin{array}{cccc} Weight & of & dry & starch\times 100\% \end{array} \end{array}$$

$$SP\;=\;\begin{array}{cccc} Weight & of & se\;\text{dim}\;ent & /\;\begin{array}{cccc} Weight & of & dry & starch\times \left(100-S\right)\% \end{array} \end{array}$$

### Statistical analysis

All experiments were done in triplicate and statistical analysis was done with SAS version 9.2 (SAS Institute, Inc., Cary, NC) to determine difference of means using Duncan’s multiple range test functions. Differences were considered to be statistically significant at *p* < 0.05. All data were expressed as mean ± standard deviation.

## Results and Discussion

### Enzymatic hydrolysis

**Fig 1:**
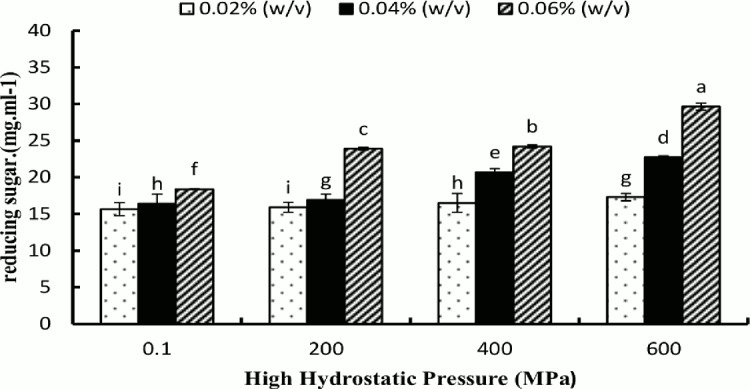
The reducing sugar produced during amylase hydrolysis for 48 h at 25°C after high hydrostatic pressure treatment at 0.1, 200, 400, and 600 MPa with 0.02%, 0.04% and 0.06% (w/v) α-amylase.

During hydrolysis with α-amylase, the α-(1→4) linkages of the interior of polymeric starch chains were cleaved and produced simple sugars as further hydrolysis products of maltose, maltotriose and α-dextrins. The varying level of HHP influenced the action of the enzyme at different concentrations on granular starch (Fig 1 and Table 1). The reducing sugar contents and percentage of hydrolysis of hydrolyzed native potato starch (hNPS) with 0.02% (w/v) α-amylase had the lowest values, 15.64 mg mL^–1^ and 16.53%, respectively. Hydrolyzed starch with HHP_600_ by 0.06% (w/v) α-amylase yielded the greatest amount of reducing sugar and the highest percentage of hydrolysis, 29.64 mg mL^–1^ and 49.36%, respectively. Using an increased enzyme concentration with hNPS and HHP hydrolyzed starches increased the yield of reducing sugar and the percentage hydrolysis. This observation was based on an increased amount of glucose released as a result of the cleavage of α-(1→4) glycosidic bonds. In addition, owing to the effect of high pressure on the starch granule, some hydrogen bonds in the amorphous area were broken and the amorphous growth ring regions were hydrated; therefore, amorphous lamellae were formed in the crystalline domain [26] and hydrolysis of starch granules was increased with increasing levels of pressure and enzyme concentration. Furthermore, high pressure can change the ordered and amorphous structure in starch granules; for example, at 600 MPa starch granules were partially gelatinized, allowing easier accessibility for the enzyme to act on the starch granule. The increase in reducing sugar content might be due to the increased effects of enzyme and HHP treatment.

**Table 1:** Percentage hydrolysis of starches subjected to α-amylase hydrolysis with different enzyme concentration after HHP treatment.

HHP (MPa)	Enzyme (%)	Hydrolysis(%)^a)^
0.1^b)^	0.02%	16.53±0.10g
	0.04%	17.86±0.20fg
	0.06%	20.73±1.80 fde
200	0.02%	17.80±2.10fg
	0.04%	21.10±2.82 de
	0.06%	27.46±0.10 c
400	0.02%	18.30±0.05 fge
	0.04%	22.00±2.00d
	0.06%	28.02±1.98 c
600	0.02%	28.80±2.00 c
	0.04%	36.90±2.21b
	0.06%	49.36±2.55a

a) Means in a column with same letters are not significantly different at *P≤0*.*05*

b) 0.1 MPa is atmospheric pressure implying starch was only hydrolyzed by enzyme hence called hNPS.

We found a significant difference between hNPS and HHP-treated hydrolyzed starch at all three concentrations of enzyme (Table 1). The outer structure of PS is more ordered in the outer regions [27], which might be the reason, at least in part, why the hydrolyzed native potato starch (hNPS) without pre-HHP treatment showed greater resistance to enzymatic hydrolysis compared to HHP-treated starch.

### Microstructure and granule size distribution

**Fig 2:**
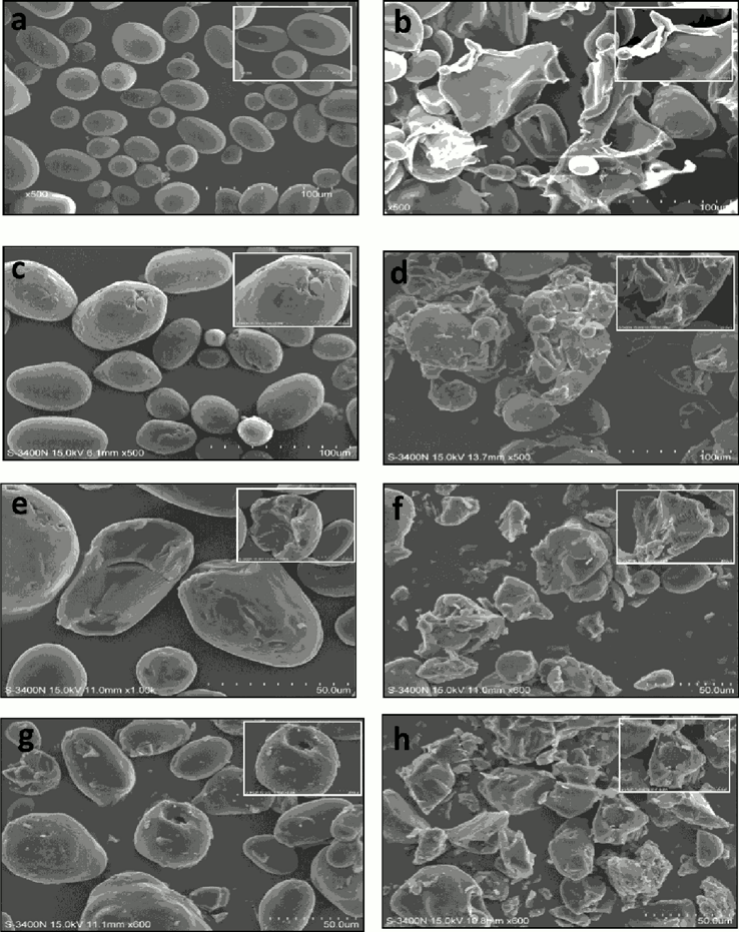
Scanning electron micrographs for native (NPS), hydrolyzed native potato starch (hNPS) and 600 MPa treated potato starches (PS) with 0.02%, 0.04% and 0.06% (w/v) α-amylase: (a) NPS, (b) 600 MPa, (c) 0.1 MPa, 0.02% (w/v), (d) 600 MPa,0.02% (w/v), (e) 0.1 MPa, 0.04% (w/v), (f) 600 MPa, 0.04% (w/v), (g) 0.1MPa, 0.06% (w/v), (h) 600 MPa, 0.06% (w/v).

The shape and granule size of various modified starches are given in Table 2 and Fig 2, respectively. Starches that were treated with HHP showed an increase in granule size with increasing pressure. Indeed, starch treated at 600 MPa had the greatest average diameter (*D*_50_) (29.43±0.1 μm) and diameter range (1.45–130.37 μm). These findings could be attributed to the partial gelatinization of starch granules. PS, which has a B-type pattern and a greater resistance to pressure compared to other starches [28], was not gelatinized completely by HHP in the range 200–600 MPa. In addition, *D*_50_ of the starch granules decreased with increase in enzyme concentration. This could be attributed to the degradation of starch granules after enzyme digestion. For better understanding and monitoring the extent of hydrolysis, SEM photographs of native, HHP-treated, native hydrolyzed and hydrolyzed HHP_600_ potato starch are shown in Fig 2. NPS had a typical elliptical and spherical shape with a smooth granule surface without pores, cracks or fissures, consistent with the report by Kaur et al. [29]. After hydrolysis, starch granules showed degradation of the external parts caused by enzymatic digestion (Fig 2C, 2E and 2G). The extent of degredation increased with increased enzyme concentration. Treatment with HHP partially gelatinized the starch granules making them more susceptible to enzymatic attack compared to native forms (Fig 2D, 2F and 2H).

**Table 2:** Granule size distribution of the native, native hydrolyzed and hydrolyzed HHP treated (200, 400, and 600 MPa) potato starches with different α-amylase concentration ^a^.

HHP (MPa)	Enzyme (%)	Diameter Range (μm)	D10 (μm)^b^	D50 (μm)^c^	D98 (μm)^d^
Native^e)^	/	2.00‒76.33	10.33±0.04ab	26.78±0.10b	65.28±0.50fde
0.1^f)^	0.02%	2.23‒94.56	10.21±0.06cab	25.62±0.40c	60.46±0.20fg
	0.04%	248‒76.33	9.65 ±0.30 c	25.77±0.50c	66.32±3.90de
	0.06%	1.45‒105.24	7.80±0.02f	23.79±0.09f	69.06±0.03dc
200	0.0%	2.23‒94.58	10.76±0.00 a	26.68±0.02b	64.72±0.01fde
	0.02%	2.23‒76.33	10.06±0.04 cb	24.31±0.10de	57.30±0.30g
	0.04%	1.62‒84.96	8.95±0.20 d	24.49±0.40d	64.68±0.60fde
	0.06%	1.45‒105.24	8.30±0.05 fe	23.69±0.06f	64.55±0.02fde
400	0.0%	2.23‒94.58	9.80±0.10 cb	26.12±0.02c	67.42±0.080d
	0.02%	2.23‒84.96	10.06±0.01cb	24.25±0.20de	60.66±0.09fge
	0.04%	2.00‒68.58	8.73±0.07de	23.97±0.02fde	65.19±3.02fde
	0.06%	1.62‒94.56	8.08±0.20 f	23.54±0.07f	65.76±0.60fde
600	0.0%	1.45‒130.37	8.37±0.77 fe	29.43±0.10a	97.88±9.60a
	0.02%	2.48‒105.24	10.77±0.40 a	28.98±0.40a	98.98±0.40a
	0.04%	1.45‒117.13	8.86±0.20de	26.88±0.20b	79.03±0.90b
	0.06%	0.62‒117.13	5.00±0.02g	20.03±0.16g	74.38±0.60bc

a) Means in a column with same letters are not significantly different at *P≤0*.*05*

b) D10 representing 10% of total particle size distribution.

c) D50 representing 50% of total particle size distribution.

d) D98 representing 98% of total particle size distribution.

e) Native potato starch (NPS)

f) 0.1 MPa is atmospheric pressure implying starch was only hydrolyzed by enzyme hence called hNPS.

### DSC analysis

The gelatinization parameters for native, HHP-treated, native hydrolyzed and hydrolyzed HHP-treated PS are given in Table 3. The onset temperature (*T*_o_) ranged between 52.42°C (for hydrolyzed HHP_600_ PS with 0.06% (w/v) α-amylase) and 58.37°C (for hNPS with 0.06% (w/v) α-amylase). The peak temperature (*T*_p_) ranged between 57.59°C (for PS HHP-treated at 400 MPa) and 63.19°C (for hydrolyzed HHP_600_ PS with 0.06% (w/v) α-amylase). Gelatinization enthalpies (Δ*H*_gel_) ranged between 4.41 J g^–1^ (for hydrolyzed HHP_600_ PS with 0.06% (w/v) α-amylase) to 10.92 J g^–1^ (for hNPS with 0.06% (w/v) α-amylase). Compared with NPS, a reduction was found in gelatinization temperatures and enthalpy with increasing pressure treatment, which was accordance with the results of lotus seed starch treated with HHP [30]. Starch could be gelatinized by HHP treatment when dispersed in the excess water. It was reported that when starch dispersed in the excess water the HHP-induced starch gelatinization had two stages, at the first stage, the changing of starch granule morphology could not be observed, but once the pressure up to a specific level the starch granule would beyond the endpoint of the first stage and reach the second stage in which starch granules will be disintegrated [31]. For hNPS, the transition temperatures (*T*_o_ and *T*_p_) and the enthalpies of gelatinization (Δ*H*_gel_) shifted to higher values with increasing enzyme concentration. Two events occur during endothermic transition First, gelatinization, primarily water molecules diffuse freely into the amorphous region of starch. Second, they penetrate the crystalline region [32]. Enzymatic treatment of NPS could have had more effect on the amorphous region compared to the crystalline region, thus delaying gelatinization, which requires a higher temperature to initiate the gelatinization process, leading to a lower gelatinization temperature range. Conversely, hydrolyzed HHP-treated starch displayed different behavior. These results could be attributed to increased high pressure and enzyme concentration since the degree of crystallinity decreased. Treatment with HHP disrupted the organization of the starch granules, leading to easy accessibility of the crystalline region of the granule to water resulting in a lower gelatinization temperature. In addition, increasing pressure caused partial gelatinization, so the crystalline structure and molecular order had been lost. Similar results have been reported for waxy maize (*Zea mays* var. ceratina), maize (*Zea mays* var. indentata), rice (*Oryza sativa*), potato (*Solanum tuberosum*) starch and wheat (*Triticum aestivum*) starch [11].

**Table 3:** Gelatinization parameters of native, native hydrolyzed and hydrolyzed HHP treated (200, 400, and 600 MPa) potato starches with different α-amylase concentration ^a^.

HHP (MPa)	Enzyme (%)	T_o_ (°C)	T_p_ (°C)	T_c_ (°C)	ΔH_gel_ (J/g)	R (°C)
Native^b)^	/	57.09±0.01a	60.40±0.05gh	62.09±0.06a	10.75±0.02a	6.62±0.01k
0.1^c)^	0.02%	57.66±0.05a	60.86±0.02j	61.41±0.02b	10.47±0.06ab	6.40±0.02l
	0.04%	57.79±0.10b	60.33±0.06h	60.58±0.10e	10.16±0.09c	4.73±0.05m
	0.06%	58.37±0.06b	60.97±0.02e	59.11±0.05i	10.92±0.06d	4.84±0.02m
200	0.0%	54.03±0.01cde	57.95±0.01l	62.18±0.02a	10.24±0.05cb	7.84±0.01j
	0.02%	54.38±0.01c	60.73±0.01f	61.09±0.02c	10.23±0.05c	12.70±0.01g
	0.04%	53.74±0.05ef	60.78±0.03f	57.87±0.03k	9.68±0.02e	14.80±0.05d
	0.06%	53.81±0.10ef	60.45±0.10g	58.71±0.10j	7.70±0.10g	13.28±0.10f
400	0.0%	54.26±0.02cd	57.59±0.02m	59.34±0.02h	10.14±0.05cd	6.66±0.02k
	0.02%	54.23±0.02cd	60.09±0.02i	58.94±0.02i	9.54±0.05e	11.72±0.02h
	0.04%	53.90±0.06h	61.21±0.05d	60.61±0.05d	9.07±0.09f	14.62±0.02e
	0.06%	53.20±0.10hg	61.85±0.09c	59.96±0.05f	6.18±0.05h	17.30±0.06b
600	0.0%	53.39±0.01hg	58.51±0.03k	59.63±0.02g	6.11±0.02h	10.24±0.01i
	0.02%	53.60±0.10efg	61.83±0.03c	60.62±0.02d	5.43±0.09i	16.64±0.01c
	0.04%	53.51±0.20hfg	62.15±0.09b	60.59±0.10d	5.19±0.06j	17.28±0.09b
	0.06%	52.42±0.20i	63.19±0.06a	61.11±0.10c	4.41±0.30k	21.54±0.05a

*T*_*o*_ onset transition temperature; *T*_*p*,_ peak transition temperature, *T*_*c*,_ conclusion transition temperature, R, gelatinization range 2(*T*_*p-*_ *T*_*o)*,_ *ΔH*_*gel*,_ enthalpy of gelatinization.

**a)** Means in a column with same letters are not significantly different at *P≤0*.*05*

**b)** Native potato starch (NPS)

**c)** 0.1 MPa is atmospheric pressure implying starch was only hydrolyzed by enzyme hence called hNPS.

The difference in *R*-value between the samples suggested a transformation in the crystalline regions of the starch granules [33]. Gunaratne & Hoover [34] suggested variability of the *R*-value for the various starch types might be owing to the differences in the strength of heterogeneous crystals.

Overall, the decrease in transition temperatures and enthalpies of gelatinization of the hydrolyzed HHP-treated samples demonstrated a reduction in the amorphous area of the granules, which was accompanied by a change in the crystalline regions following a cooperative process in the case of HHP, but led to more crystalline structure in the hNPS starches.

### FTIR analysis

**Fig 3:**
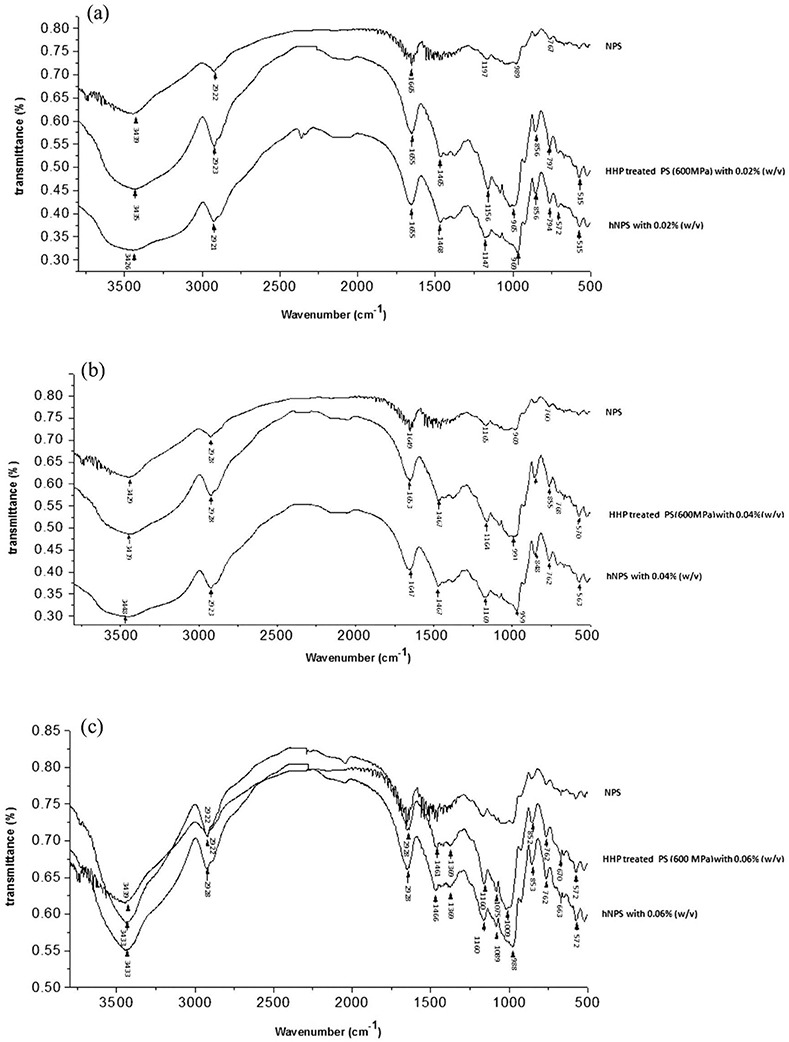
FTIR spectrum of native (NPS), hydrolyzed native potato starch (hNPS) and 600 MPa treated potato starches (PS) with 0.02%, 0.04% and 0.06% (w/v) α-amylase. a: 0.02% (w/v) α-amylase; b: 0.04% (w/v) α-amylase; c: 0.06% (w/v) α-amylase.

FTIR spectra of native, native hydrolyzed and hydrolyzed HHP_600_ potato starch are presented in Fig 3. Spectral changes can be divided into band narrowing and changes in the absorption intensity of specific bands [35]. Band narrowing was caused by ordering of the polymers and a reduction in the number of conformations; change of band intensity resulted from change of specific conformations, such as long-range ordering and crystallinity [36]. The broad bands from 3100–3700 (cm^–1^) represent complex vibrational stretches associated with intermolecular hydroxyl groups [37] The native, hydrolyzed native and hydrolyzed HHP_600_ PS with 0.02% and 0.04% (w/v) α-amylase showed the same broad bands from 3100–3700 cm^–1^ (Fig 3A and 3B), while hydrolyzed native and hydrolyzed HHP_600_ PS with 0.06% (w/v) α-amylase showed a greater intensity (Fig 3C). The sharp bands at 2920–2928 cm^–1^ were related to the CH_2_ vibration [38], as shown in Fig 3C for the native hydrolyzed and hydrolyzed HHP-treated potato starch with 0.06% (w/v) α-amylase. This could be attributed to changes in conformation and crystallinity. The peak at ~1640 cm^–1^ was a sign of firmly bound water present in the native, enzyme-treated and hydrolyzed HHP-treated starch under different conditions. Compared to native starch, peak height was increased but there was no significant change of peak width after hydrolysis and treatment with HHP. The absorption band at ~1458 cm^–1^ was attributed to compactly bound water present in starch [39], of which significant changes were observed for all three treatment conditions compared to NPS. The intensity at 920~980 cm^–1^ was attributed to the skeletal mode vibrations of α-(1→4) glycosidic linkage (C-O-C). The peaks at 1081, 1156, and 1020 cm^–1^ were characteristic of the anhydrous glucose ring C-O stretch [38]. The band at ~850 cm^–1^ was sensitive to changes of crystallinity [35]. Compared to NPS, the native hydrolyzed and hydrolyzed HHP-treated potato starches with 0.02% and 0.04% (w/v) α-amylase showed no significant change of peak position or intensity but exhibited significant changes in the fingerprint area. Peaks became sharper with increased enzyme concentration and some bands were absent or not pronounced in the NPS spectrum, suggesting significant changes in both the ordered and the amorphous structures of PS.

### XRD analysis

**Fig 4:**
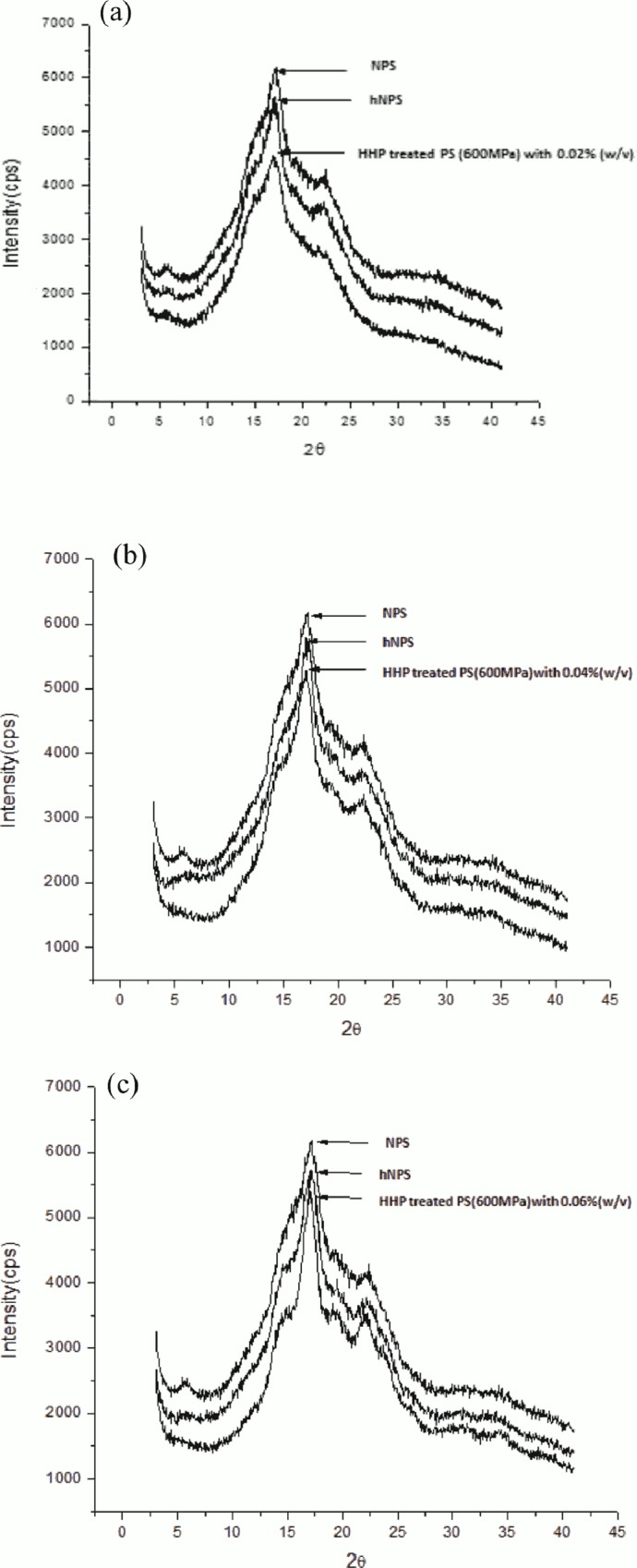
XRD pattern of native, hydrolyzed native and 600 MPa treated potato starches (PS) with 0.02%, 0.04% and 0.06% (w/v) α-amylase. a: 0.02% (w/v) α-amylase; b: 0.04% (w/v) α-amylase; c: 0.06% (w/v) α-amylase.

The XRD pattern of NPS, native hydrolyzed and hydrolyzed HHP_600_ PS are presented in Fig 4. They show a typical B-type X-ray diffraction pattern with strong peaks at 2θ of 5.7°, 17.1° and 22.5° [40], HHP-treated potato starch with 0.6% α-amylase was an exception, with an extra weak peak at 2θ = 19.28° characteristic of a V-type crystallite. The peaks of native hydrolyzed starches became sharper with increased enzyme concentration, indicating the amorphous parts of the starch granules had been disrupted [41]. The hydrolyzed HHP_600_ starches, however, displayed different behavior. The intensity of the peaks was changed slightly owing to the loss of crystallinity during treatment with HHP [42]. The small peak at 5.6° disappeared, while the peaks at ~19° and ~15° became larger (Fig 4B and 4C). An additional peak at 2θ = 19.36° indicated the crystal of HHP_600_ potato starch with 0.06% (w/v) α-amylase was in the transformation from B type to B+V type with a majority of B-type crystallite. It might be owing to an increased proportion of short amylose chains, which tend to form double helices [43]. In addition, α-amylase can penetrate the starch granule more efficiently and change the organized structure of granules pretreated with HHP compared to hydrolyzed NPS. These results indicated amylolysis occurs in both the amorphous and the crystalline regions of starch granules.

### Swelling power and solubility

**Fig 5:**
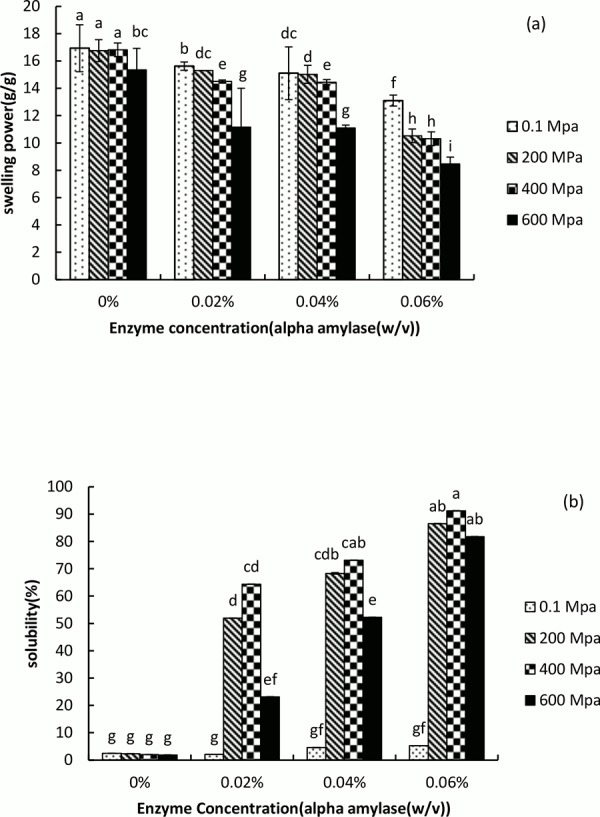
Swelling power (a) and Solubility (b) of native, hydrolyzed native and 200, 400, and 600 MPa treated potato starches (PS) with 0.02%, 0.04% and 0.06% (w/v) α-amylase.

The swelling power and solubility of NPS, hydrolyzed NPS, HHP-treated and hydrolyzed HHP-treated PS are presented in Fig 5. A decrease of swelling power was observed for HHP-treated and hydrolyzed HHP-treated PS with different concentrations of enzyme (Fig 5A). Compared to NPS, the swelling power of HHP-treated PS was decreased from 16.93 to 15.35 g/g. After hydrolyzed by α-amylase, the swelling power of hydrolyzed NPS and hydrolyzed HHP-treated PS decreased significantly, among which the hydrolyzed HHP-treated PS with α-amylase at a concentration 0.06% (w/v) showed lowest swelling power, 8.47 g/g (P< 0.05). The results above might be due to that HHP and enzymatic hydrolysis could alter both the crystalline and the amorphous structure of starch granules. These results were in accord with the report by Tester et al. [44] focused on the effect of hydrolysis on the swelling behavior of starch granules. The solubility of native and hydrolyzed HHP-treated (200 and 400 MPa) samples increased with increasing α-amylase concentration. It is worth mentioning PS is more pressure-resistant compared to other starches [45] and pressures of less than 600 MPa did not have a significant effect on starch granules; hence, potato starch granules needed > 600 MPa pressure for complete gelatinization [46]. The action of α-amylase led to the conversion of amylose into low molecular mass components such as glucose and dextrin. And the characteristics of amylose and amylopectin were also changed, including the molecular mass, degree of branching, chain length and conformation. This is in accord with the study of hydrolysis and heat-treated starches at sub-gelatinization reported by Uthumporn et al. [41]. By contrast, the HHP_600_ sample hydrolyzed with different concentrations of enzyme showed decreased solubility compared to hydrolyzed HHP-treated (200 and 400 MPa) starches at different enzyme concentrations. It is likely high pressure induced formation of the amylose–lipid complex and inhibited amylose leaching, which led to decreased solubility [47].

## Conclusion

This study demonstrated the effect of HHP and enzymatic hydrolysis with α-amylase at different levels of pressure and concentration of enzyme on various physicochemical properties of potato starch. Pre-HHP treatment of starches at 600 MPa increased susceptibility to enzymatic hydrolysis with an increased amount of reducing sugar produced. Further, there was significant change of the *D*_50_ value, thermal properties and crystalline structure pattern. Swelling power and solubility decreased with increasing level of HHP, owing, at least in part, to change in the intramolecular structure of starch. The hydrolyzed HHP-treated starch, however, showed decreased swelling power but increased solubility with increasing HHP level and enzyme concentration. By contrast, hydrolyzed starches HHP-treated at 200 and 400 MPa showed little or no difference in structural, morphological or functional properties compared to the native form, which showed that potato starch with B-type pattern are resistant to high pressure, especially below 600 MPa. The information obtained from this study suggests the possibility of modifying the physicochemical, functional and morphological properties of PS without heat treatment, obviating any undesirable side effects. This modification process could be practical in the food and related industries.

## Abbreviations

NPS: native potato starch
PS: potato starch
hNPS: hydrolyzed native potato starch
HHP: high hydrostatic pressure
HHP_600_: HHP-treated (600MPa)
